# Estimates of introgression as a function of pairwise distances

**DOI:** 10.1186/s12859-019-2747-z

**Published:** 2019-04-23

**Authors:** Bastian Pfeifer, Durrell D. Kapan

**Affiliations:** 1 Institute for Medical Informatics, Statistics and Documentation, Medical University, Graz, Austria; 2 Department of Entomology and Center for Comparative Genomics, Institute for Biodiversity Science and Sustainability, California Academy of Sciences, 55 Music Concourse Dr., San Francisco, USA

**Keywords:** Genomics, Introgression, Hybridisation, SNPs

## Abstract

**Background:**

Research over the last 10 years highlights the increasing importance of hybridization between species as a major force structuring the evolution of genomes and potentially providing raw material for adaptation by natural and/or sexual selection. Fueled by research in a few model systems where phenotypic hybrids are easily identified, research into hybridization and introgression (the flow of genes between species) has exploded with the advent of whole-genome sequencing and emerging methods to detect the signature of hybridization at the whole-genome or chromosome level. Amongst these are a general class of methods that utilize patterns of single-nucleotide polymorphisms (SNPs) across a tree as markers of hybridization. These methods have been applied to a variety of genomic systems ranging from butterflies to Neanderthals to detect introgression, however, when employed at a fine genomic scale these methods do not perform well to quantify introgression in small sample windows.

**Results:**

We introduce a novel method to detect introgression by combining two widely used statistics: pairwise nucleotide diversity *d*_*xy*_ and Patterson’s *D*. The resulting statistic, the *distance fraction* (*d*_*f*_), accounts for genetic distance across possible topologies and is designed to simultaneously detect and quantify introgression. We also relate our new method to the recently published *f*_*d*_ and incorporate these statistics into the powerful genomics R-package PopGenome, freely available on GitHub (*pievos101/PopGenome*) and the Comprehensive R Archive Network (CRAN). The supplemental material contains a wide range of simulation studies and a detailed manual how to perform the statistics within the PopGenome framework.

**Conclusion:**

We present a new distance based statistic *d*_*f*_ that avoids the pitfalls of Patterson’s *D* when applied to small genomic regions and accurately quantifies the fraction of introgression (*f*) for a wide range of simulation scenarios.

**Electronic supplementary material:**

The online version of this article (10.1186/s12859-019-2747-z) contains supplementary material, which is available to authorized users.

## Background

Hybridization between species is increasingly recognized as a major evolutionary force. Although long known to occur in plants, evidence is mounting that it regularly occurs in many animal groups [1]. Generally thought to decrease differences between two species by sharing alleles across genomes, hybridization can paradoxically act as a ready source of variation, impacting adaptation [2, 3], aiding in evolutionary rescue [4], promoting range expansion [5], leading to species divergence [6, 7] and ultimately fueling adaptive radiation [8, 9]. The advent of whole genome sequencing has prompted the development of a number of methods to detect hybridization across the genome (recently summarized in Payseur and Rieseberg [10])

One class of methods involves quantifying single nucleotide polymorphism (SNP) patterns to detect hybridization between taxa. Here we focus on this class of tests involving four taxa. The most widely used of these, Patterson’s *D*, was first introduced by Green et al. [11] and further developed by Durand et al. [12]. Patterson’s *D* compares allele patterns of taxa with the Newick tree (((P1,P2),P3),O), to detect introgression between archaic taxon 3 (P3) and in-group taxon 1 (P1) or 2 (P2 or vice-versa). In brief, assuming the outgroup O is fixed for allele A, derived alleles (B) in P3, when shared with either P2 or P1, act as a marker of introgression leading to the following patterns: ABBA or BABA respectively. An excess of either pattern, ABBA or BABA represents a difference from the 50:50 ratio expected from incomplete lineage sorting and thus represents a signal that can be used to detect introgression.

Since its introduction, Patterson’s *D* has been used for a wide range of studies to estimate the overall amount of hybrid ancestry by summing the ABBA or BABA pattern excess on a whole genome scale starting with studies of Neanderthals and archaic humans [11, 12]. In the past 7 years, Patterson’s *D* has been increasingly used to localize regions of hybrid ancestry, not only in archaic humans [13] but also in species including butterflies, plants and snakes [14–16].

Currently, Patterson’s *D* is frequently used in sliding window scans of different regions of the genome [17–19]. However, intensive evaluations of the four-taxon ABBA-BABA statistics [20] showed that this approach can lead to many false positives in regions of low recombination and divergence. One of the main reasons is the presence of mainly one of the two alternative topologies as a consequence of a lack of independence of adjacent genomic regions [20], resembling an introgression event, which is exacerbated when analyzing smaller gene-regions. To circumvent this issue, several strategies have been developed. On one side, more sophisticated non-parametric methods have been used to reduce the number of false positives (e.g., Patterson et al. [21]). On the other side, new statistics have been developed to better estimate the proportion introgression. Martin et al. [20] recently proposed the *f*_*d*_ estimate based on the *f* estimates (e.g. *f*_*G*_, *f*_*hom*_) originally developed by Green et al. [11] which measure the proportion of unidirectional introgression from P3 to P2. Specifically, *f*_*d*_ assumes that maximal introgression will lead to equally distributed derived allele frequencies in the donor and the recipient population and therefore utilizes the higher derived allele frequency at each variant site. This strategy aims to model a mixed population maximally affected by introgression. However, this approach has two major shortcomings: First, it is designed to sequentially consider introgression between the archaic population P3 and only one ingroup taxa (P1 or P2). Second, the accuracy of measuring the fraction of introgression strongly depends on the time of gene-flow.

Here we combine the approaches of the four-taxon tests with genetic distance to derive a statistic, the *distance fraction* (*d*_*f*_), that estimates the proportion of introgression on a four-taxon tree which strictly ranges from -1 to 1, has symmetric solutions, can be applied to small genomic regions, and is less sensitive to variation in the time of gene-flow than *f*_*d*_.

## Approach

To derive *d*_*f*_ we took a two-fold approach. First, we reformulated Patterson’s *D*, and *f*_*d*_ in terms of genetic distances based on the hypothesis that past or recent hybridization will leave a signature of reduced *d*_*xy*_ between taxa [18, 22]. Second, we account for non-introgressed histories by incorporating distances from species tree patterns into the denominator.

First, following convention, A and B denote ancestral and derived alleles respectively. Derived allele frequencies of the four taxa are *p*_1*k*_…*p*_4*k*_ at variant site *k*. Second, *d*_*xyk*_ is the average pairwise nucleotide diversity (genetic distance) between population *x* and *y* at variant site *k*. Each genetic distance can be expressed as a sum of patterns in terms of ancestral and derived alleles allowing the terms ABBA and BABA to be rewritten in terms of genetic distances.

### Patterson’s *D* statistic as a function of pairwise distances

Here we derive the Patterson’s *D* statistic as a function of pairwise genetic distance between taxon *x* and taxon *y* (*d*_*xy*_). Following [23] the genetic distance *d*_*xy*_ is defined as

$$\begin{array}{@{}rcl@{}} d_{xyk} = \frac{1}{n_{x}n_{y}} \sum^{n_{x}}_{i=1}\sum^{n_{y}}_{j=1}\pi_{ijk}  \end{array} $$

at a given variant site *k*, where *n*_*x*_ is the number of individuals in population *x* and *n*_*y*_ is the number of individuals in population *y*. Then at site *k*, *π*_*ij*_=1∨0 is the boolean value indicating that the individual *i* of population *x* and the individual *j* of population *y* contains the same variant (0) or not (1). Following [12, 21] instead of pattern counts, allele frequencies can be used as an unbiased estimator. Given only bi-allelic sites (SNPs) the genetic distances *d*_*xy*_ can be formulated as a function of allele frequencies (*p*) as follows:

$$\begin{array}{@{}rcl@{}} d_{12k}= p_{1k}(1-p_{2k}) + (1-p_{1k})p_{2k}\\ d_{13k}= p_{1k}(1-p_{3k}) + (1-p_{1k})p_{3k}\\ d_{23k}= p_{2k}(1-p_{3k}) + (1-p_{2k})p_{3k} \end{array} $$

If we define *a* as the ancestral allele frequency (1−*p*) and *b* as the derived allele frequency (*p*) then

$$\begin{array}{@{}rcl@{}} d_{12k}= b_{1k}a_{2k} + a_{1k}b_{2k}\\ d_{13k}= b_{1k}a_{3k} + a_{1k}b_{3k}\\ d_{23k}= b_{2k}a_{3k} + a_{2k}b_{3k} \end{array} $$

Note, the fourth taxon (outgroup) is used to define the ancestral state *a*.While incorporating the species tree pattern **BBAA**, the introgression patterns ABBA and BABA can be re-written in terms of allele frequencies:

$$ \begin{aligned} {ABBA}_{k}:= & [(\mathbf{b}_{1k}\mathbf{b}_{2k}\mathbf{a}_{3k}\mathbf{a}_{4k} + a_{1k}b_{2k}b_{3k}a_{4k}) \\ & - (\mathbf{b}_{1k}\mathbf{b}_{2k}\mathbf{a}_{3k}\mathbf{a}_{4k} + b_{1k}a_{2k}b_{3k}a_{4k}) \\ & + (b_{1k}a_{2k}b_{3k}a_{4k} + a_{1k}b_{2k}b_{3k}a_{4k})]/2  \end{aligned}  $$

$$ \begin{aligned} {BABA}_{k}:= & [(\mathbf{b}_{1k}\mathbf{b}_{2k}\mathbf{a}_{3k}\mathbf{a}_{4k}+b_{1k}a_{2k}b_{3k}a_{4k}) \\ & - (\mathbf{b}_{1k}\mathbf{b}_{2k}\mathbf{a}_{3k}\mathbf{a}_{4k}+a_{1k}b_{2k}b_{3k}a_{4k}) \\ & + (b_{1k}a_{2k}b_{3k}a_{4k}+a_{1k}b_{2k}b_{3k}a_{4k})]/2 \end{aligned}  $$

Using distances (*d*_*xy*_) from above, these patterns can then be expressed as:

$$\begin{array}{@{}rcl@{}} {ABBA}_{k}=[p_{2k}\cdot d_{13k}-p_{1k}\cdot d_{23k}+p_{3k}\cdot d_{12k}]\cdot (1-p_{4k})/2 \\ {BABA}_{k}=[p_{1k}\cdot d_{23k}-p_{2k}\cdot d_{13k}+p_{3k}\cdot d_{12k}]\cdot (1-p_{4k})/2 \end{array} $$

Finally, this leads to the following distance based Patterson’s *D* equation for a region containing *L* variant positions:

1 $$\begin{array}{@{}rcl@{}} D=\frac{\sum^{L}_{k=1} {ABBA}_{k} - {BABA}_{k}}{\sum_{k=1}^{L}{ABBA}_{k}+{BABA}_{k}}=\frac{\sum^{L}_{k=1} p_{2k}\cdot d_{13k} - p_{1k}\cdot d_{23k}}{\sum_{k=1}^{L}p_{3k}\cdot d_{12k}} \end{array} $$

In the context of distances *p*_2*k*_·*d*_13*k*_ may be seen as the contribution of the variation contained between the lineages 1 to 3 (*d*_13*k*_) to population 2.

As seen from Eq. (1) the Patterson’s *D* denominator (ABBA + BABA) simplifies to an expression of the derived allele frequency of the archaic population P3 times the average pairwise nucleotide diversity (*d*_*xy*_) between population P1 and P2. This interpretation highlights the original difficulty that Patterson’s *D* has handling regions of low diversity since the denominator will be systematically reduced in these areas due to the *d*_12*k*_ variable; increasing the overall *D* value. This effect intensifies when at the same time the divergence from the donor population P3 is high. Martin et al. [20] proposed *f*_*d*_ which corrects for this by considering the higher derived allele frequency (P2 or P3) at each given variant position; systematically increasing the denominator.

### Martin’s *f*_*d*_ estimator

We can apply the same distance logic to rewrite the *f*_*d*_ statistic. Following the example above for *D* we start with the definition of the statistic *f*_*hom*_ [11] upon which *f*_*d*_ is based. The basic idea of the *f*_*hom*_ estimate is that complete introgression would lead to complete *homogenization* of allele frequencies. Here it is assumed that introgression goes from P3 to P2, therefore:

$$\begin{array}{@{}rcl@{}} f_{hom} = \frac{S(P1,P2,P3,O)}{S(P1,P3,P3,O)} \end{array} $$

where the numerator is the same as Patterson’s *D*:

$$\begin{array}{@{}rcl@{}} S(P1,P2,P3,O)=\sum_{k}^{L}p_{2k}\cdot d_{13k}-p_{1k}\cdot d_{23k} \end{array} $$

and the denominator can be formulated by substituting P2 with P3,

$$\begin{array}{@{}rcl@{}} S(P1,P3,P3,O)=\sum_{k}^{L}p_{3k}\cdot d_{13k}-p_{1k}\cdot \pi_{3k}  \end{array} $$

where *π*_3*k*_ is the average pairwise nucleotide diversity within population P3 at site *k*. The terms *p*_3*k*_·*d*_13*k*_ may be interpreted as the contribution of population 3 to the variation contained between the lineages 1 to 3 (subtracting the contribution of population 1 contained in population 3). Following Martin et al. [20] *f*_*d*_ is defined as $f_{d} = \frac {S(P1,P2,P3,O)}{S(P1,PD,PD,O)}$ where PD is the population (2 or 3) with the higher derived allele frequency at each variant position. Here the denominator is:

2 $$\begin{array}{@{}rcl@{}} S(P1,PD,PD,O)&\,\,\,=\sum_{k}^{L}p_{Dk}\cdot d_{1Dk}-p_{1k}\cdot d_{DDk}\\&=\sum_{k}^{L}p_{Dk}\cdot d_{1Dk}-p_{1k}\cdot \pi_{Dk} \end{array} $$

Leading to the statistic:

3 $$\begin{array}{@{}rcl@{}} f_{d}=\frac{\sum_{k=1}^{L}{p}_{2k}\cdot d_{13k} - {p}_{1k}\cdot {d}_{23k} }{\sum_{k=1}^{L}{p}_{Dk}\cdot {d}_{1Dk}-p_{1k}\cdot \pi_{Dk}} \end{array} $$

where in the denominator, *π*_*Dk*_ is the nucleotide diversity within population PD, which is the population with the higher derived allele frequency (P2 or P3) for each variant site *k*. The difference between the *f*_*hom*_ statistic versus *f*_*d*_ is that there is no assumption in the latter about the direction of introgression.

**Fig. 1:**
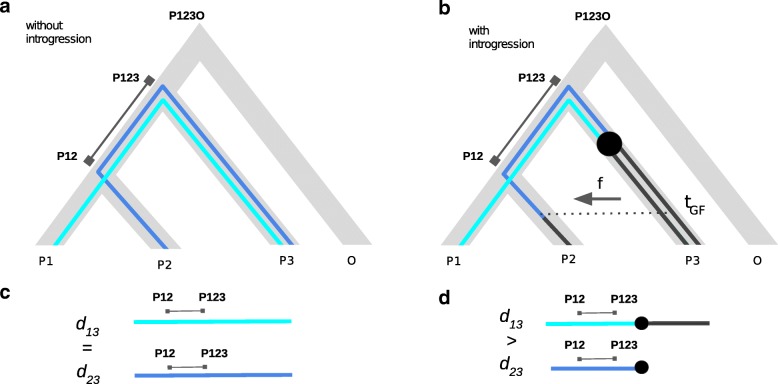
A graphical interpretation of the *d*_*f*_ estimate. The distance fraction (*d*_*f*_) estimates the fraction of introgression (f) by relating the differences of the genetic distances between taxa, here hi-lit by path lengths between ingroup taxa (*d*_13_= light blue, *d*_23_= dark blue) to the overall sum of the path lengths to the archaic population P3 taking into account derived alleles resulting in a change of path length distance. a. The four-taxon (P1, P2, P3 and O) species tree (gray) with coalescence at nodes denoted as P12, P123 and P123O. Path length P12-P123 helps visualize the scale of relative distance between taxa and signifies the shared distance of P1 and P2 to P3. b. Illustrates introgression from P3 to P2, here marked by derived alleles arising in and replacing the P3 lineage after the split leading to P12 (black dot). c. Without introgression *d*_13_=*d*_23_ and resulting in *d*_*f*_=0 (left a & c). d. Introgression of derived alleles reduces genetic distance between P2 and P3 at the time of gene-flow (*t*_*GF*_) causing *d*_23_<*d*_13_ and *d*_*f*_ to be positive (right b & d). Note, allele replacement in example (b, d) corresponds to SNP pattern ABBA. The *d*_*f*_ estimate relates the reduced distance caused by introgression to the total sum of path length distances after introgression. A mutation on the P12-P123 path corresponds to the SNP pattern BBAA and signifies shared distance

The distance based interpretations (above) for SNP based introgression statistics Patterson’s *D* and *f*_*d*_ suggest that it would be beneficial to derive estimators for the proportion of introgression that are free from the problem of reduced diversity. Here we propose a very simple statistic we call the *distance fraction* (*d*_*f*_), that makes direct use of the distance based numerator of the Patterson’s *D* statistic and relates the differences of distances to the total distance considered (Fig. 1) by incorporating the BBAA species tree pattern into the denominator. The species tree pattern BBAA contributes to increased divergence between (P1,P2) and P3 in the absence of introgression. As a consequence within our *d*_*f*_ framework, we explicitly include the divergence to P3 on the four-taxon tree.

### The *d*_*f*_ estimator

In distance terms we may interpret the ABBA and BABA patterns as polarized shared distances (shared distance between two taxa caused by the derived alleles) on a 4-taxon tree. ABBA for example can be interpreted as the polarized shared distance between (P2,P3) and P1, where BABA is the polarized shared distance between (P1,P3) and P2. Thus, ABBA is a signal of shared increased distance to P1 and BABA is a signal of shared increased distance to P2. This leads naturally to the distance based numerator that is the same as Patterson’s *D* statistic Eq. (1).

However, for the denominator, in order to relate those distances to the distances which are not a signal of introgression, the BBAA pattern must to be taken into account, because the species tree captures the third way in which exactly two populations can share derived alleles. According to the interpretations given above, the BBAA species tree pattern can be seen as the polarized shared distances of (P1,P2) to P3. We incorporate this pattern to refine two classes given the system described above:

- **Class 1**: The contribution of derived alleles in P2 to distance (ABBA+BBAA).
- **Class 2**: The contribution of derived alleles in P1 to distance (BABA+BBAA).

The union of both classes includes all possible patterns producing distances on a 4-taxon tree by shared derived alleles. Thus, to incorporate all these distances, those representing the BBAA pattern must be added to the denominator, *d*_*f*_ can be written as:

4 $$\begin{array}{@{}rcl@{}} &&\sum_{k=1}^{L}({ABBA}_{k}+{BBAA}_{k})+({BABA}_{k}+{BBAA}_{k})\\&&=\sum_{k=1}^{L}p_{2k}\cdot {s}_{13k} + {p}_{1k}\cdot {d}_{23k} \end{array} $$

For a given region including *L* variant sites.A decreased BBAA polarized shared distance and an increased polarized shared distance ABBA is a signal of *P*3⇔*P*2 introgression. When at the same time the BABA signal reduces we have a maximal support for the ABBA signal.

To hi-light the exclusive distances due to introgression the *d*_*f*_ statistic we propose here has the following form:

5 $$\begin{array}{@{}rcl@{}} {d}_{f}=\frac{\sum_{k=1}^{L}{p}_{2k}\cdot {d}_{13k} - p_{1k}\cdot {d}_{23k}}{\sum_{k=1}^{L}p_{2k}\cdot {d}_{13k} + {p}_{1k}\cdot {d}_{23k}} \end{array} $$

In distance terms, *d*_*f*_ may be interpreted as the difference of the distances from P1 and P2 to the archaic population P3 that is caused by introgression (Fig. 1). The transformation of the denominator back into the basic Patterson’s *D* statistic form suggests adding the given species tree BBAA pattern to the ABBA and BABA class respectively; which can be reasonably assumed to be the most likely pattern in the absence of introgression for a given species tree (((P1,P2),P3),O). With these patterns in hand it becomes possible to distinguish between signals of introgression and non-introgression. It should be noticed, however, that the *d*_*f*_ equation still produces some extreme values when e.g the derived allele frequency *p*_1_ or *p*_2_ is zero (often true when block-size is small). To mitigate this issue, we encourage the user to apply *Laplace smoothing* in genomic scan applications. In this case the derived allele frequency *p* is simply replaced by $p=\left (\sum _{k=1}^{n+2}\pi +1\right)/(n+2)$ for population P1 and P2 and *d*_*xy*_ is updated accordingly. The parameter *π* is a boolean variable and equals to 1 when a derived allele is present. Thus, we simply add a derived allele and an ancestral allele to the populations P1 and P2. We have implemented *Laplace smoothing* for *d*_*f*_ as a feature in PopGenome.

### Simulation study

To evaluate the performance of the *d*_*f*_ we used a simulation set-up following Martin et al. [20]. The Hudson’s ms program [24] was used to generate the topologies with different levels of introgression and the seq-gen program [25] to generate the sequence alignments upon which to compare the performance of the three main statistics discussed in this paper, Patterson’s *D* (*D*), *f*_*d*_ and *d*_*f*_. The baseline simulation is shared with [20] and is performed as follows:

ms 32 1 -I 4 8 8 8 8 -ej 1 2 1 -ej 2 3 1 -ej 3 4 1 -es 0.1 2 0.9 -ej 0.1 5 3 -r 50 5000 -T | tail -n + 4 | grep -v // > treefile

The above Unix call produces the trees and stores them into a file (*treefile*). Next, we will store the number of trees in an object called *partitions*.

partitions=($(wc -I treefile))

With these parameters as an input we are now able to call the *seq-gen* program to generate the actual sequences and we store the results into a file called *seqfile*.

seq-gen -mHKY -I 5000 -s 0.01 -p $partitions < treefile > seqfile

These example calls generate a 5kb sequence with 8 samples for each of the four populations (-I) with split times P12= 1×4*N*, P123= 2×4*N* and P123O= 3×4*N* generations ago (-ej). The time of gene-flow (*t*_*GF*_) is set to 0.1×4*N* generations ago with a fraction of introgression of *f*=0.1 (-es). The recombination rate is *r*=0.01 (-r) and the Hasegawa-Kishino-Yano model substitution model was applied with a branch scaling factor of *s*=0.01 (-s). Note, we have created a GitHub repository (*pievos101/Introgression-Simulation*) including more example calls and add the option to use the R-package PopGenome to directly apply the proposed statistics to simulated datasets.

Simulations were varied across a wide range of parameters such as distance to ancestral population, time of gene flow, recombination, ancestral population size and the effect of low variability, window size and sample size as detailed in the Additional file 1: Section S1. These simulations had the following in common: for each fraction of introgression *f*=[0,0.1,…,0.9,1], we simulated 100 loci, we calculated *D*, *f*_*d*_ and *d*_*f*_ and assessed their performance with three standard statistics: adjusted *R*^2^ (a measure of the ’goodness of fit’), the ’sum of squares due to lack of fit’ (SSLF) the sum of squared distances from the mean value for each fraction of introgression estimated to the real fraction of introgression, and the ’pure sum of squares error’ (SSPE) the sum of squared distances between each simulated value and the mean value for that simulation.

It should be noted that we simulate *P*2⇔*P*3 introgression to be able to compare the results of the proposed *d*_*f*_ method with the *f*_*d*_ estimate. However, *d*_*f*_ can naturally measure the fraction of introgression in both directions; with *P*2⇔*P*3 introgression *d*_*f*_ indicated by positive values (e.g. Fig. 1, change in distance due to shared ABBA pattern) and in the case of *P*1⇔*P*3 introgression negative values (BABA, not illustrated). Thus, assessing the accuracy in case of *P*2⇔*P*3 introgression applies also for *P*1⇔*P*3 introgression.

To further test *d*_*f*_, we evaluated the performance to detect introgression by simulating 10,000 neutral loci (*f*=0) and 1000 loci subject to introgression (following the parameters outlined in the above example). We interpreted the results using a receiver operating characteristic curve (ROC) analysis that evaluates the area under the curve (AUC), a measure that summarizes model performance, the ability to distinguish introgression from the neutral case, calculated with the R-package pROC [26].

We also show the application of our method to real data by calculating *d*_*f*_ for 50 kb consecutive windows on the 3L arm of malaria vectors in the *Anopheles gambiae* species complex [17]. In order to detect chromosome-wide outliers we tested the null hypotheses (*d*_*f*_=0)*outside* of the inversion, and *inside* the inversion $\left (d_{f}=\overline {d_{f}}\right)$ since the inversion was previously identified as a negative outlier [17]. The analysis was done using a weighted block jackknife to generate Z-values. The corresponding *P* values were corrected for multiple testing using the Benjamini-Hochberg false discovery rate (FDR) method [27]. This analysis is easily replicated by following the example in the Additional file 1: Section S2.

All of these analyses were done in the R-package PopGenome [28], that efficiently calculates *d*_*f*_ (and other statistics including *f*_*d*_, the recently published two-taxon *RNDmin* method [29] and the original Patterson’s *D*) from the scale of individual loci to entire genomes.

## Results

We performed extensive simulations varying distance to ancestral populations, time of gene flow, recombination, ancestral population size, the effect of low variability, window size and sample size. We found that *d*_*f*_ outperforms or is essentially equivalent to the *f*_*d*_ estimate to measure the real fraction of introgression for most of the studied ranges of simulation cases. Overall, because it captures natural variation in the denominator, *d*_*f*_ has slightly higher variances compared to *f*_*d*_ while the mean values are often the least biased as shown by the sum of squares due to lack of fit, yet it provides the best (or nearly equivalent) estimates to *f*_*d*_ as judged by the goodness of fit in almost all cases (Additional file 1: Section S1).

### The effect of background history and ancestral population sizes

**Fig. 2:**
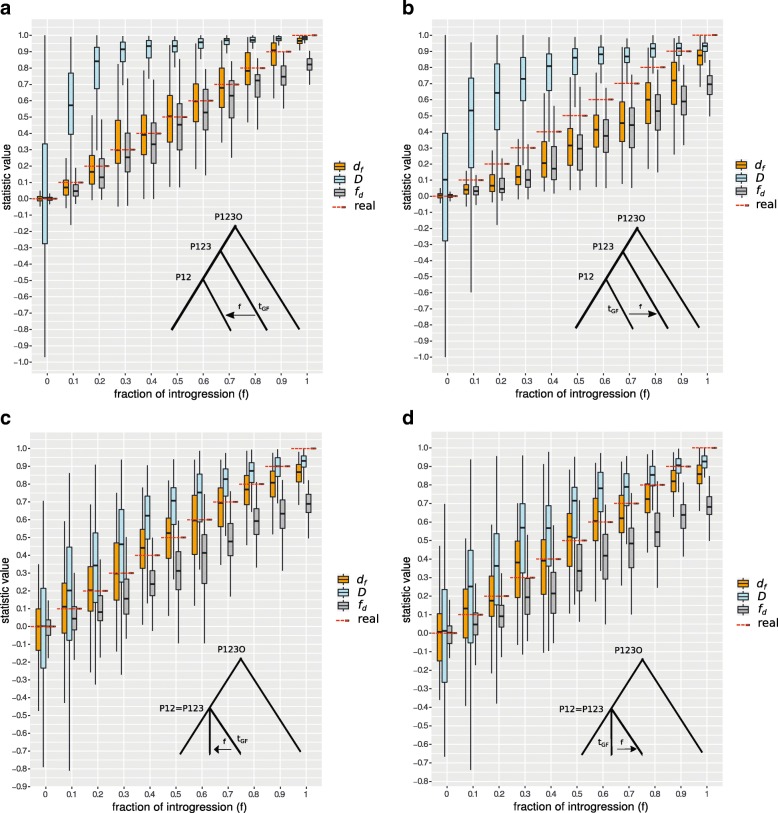
Accuracy of statistics to measure the fraction of introgression. The comparison of simulated data with a known fraction of introgression using ms versus the statistics (y-axis). We simulated 100 loci for every fraction of introgression *f*=[0,0.1,…0.9,1] and plotted the distribution of the corresponding statistic outcomes. A window size of 5kb and a recombination rate of r=0.01 was used. The background histories (coalescent events, see insets) are **a** P12= 1×4*N*, P123= 2×4*N*, P123O= 3×4*N* generations ago. **b** P12= 1×4*N*, P123= 2×4*N*, P123O= 3×4*N* generations ago. **c** P12= 1×4*N*, P123= 1×4*N*, P123O= 3×4*N* generations ago. **d** P12= 1×4*N*, P123= 1×4*N*, P123O= 3×4*N* generations ago. Introgression directions are *P*3→*P*2 (a,c) and *P*2→*P*3 (b,d) *t*_*GF*_=0.1×4*N* generations ago. Colors: *f*_*d*_ (grey), *d*_*f*_ (orange) Patterson’s *D* (light blue) and the real fraction of introgression (red dashed lines). The calls to the ms program can be found in the caption of Additional file 1: Table S1.1

Simulations under a variety of distances to ancestral populations (coalescent times) show that *d*_*f*_ is the most accurate estimator for the real fraction of introgression, including under the different coalescent events simulated for both directions of introgression (Fig. 2, Table 1). Following behind *d*_*f*_ is *f*_*d*_, which is more affected by differences in coalescent times. In this comparison, Patterson’s *D* consistently overestimates the fraction of introgression (Fig. 2, Table 1). This known effect [20] is greatest in the most common case where the coalescent times differ between ingroup taxa (P1,P2) and the archaic taxon P3 (Fig. 2a and b). This effect is also slightly impacted by the direction of introgression (e.g. lowered for *P*2→*P*3 introgression, see Fig. 2b and d, Table 1). However, for the case where the ingroup taxa (P1,P2) and the archaic taxon P3 are evolutionary very close, it should be noted that *d*_*f*_ essentially differs from the *f*_*d*_ estimate (Table 1 and Additional file 1: Table S1.1). In this specific case the SSPE of *d*_*f*_ increases leading to a lower ’goodness of fit’ compared to *f*_*d*_, while the SSLF are still notably low signifying a very precise mean estimate of the real fraction of introgression. In an further analyses we varied the ancestral population size (Additional file 1: Table S1.2). We observe that an increasing size of the ancestral population of P1 and P2 (N12) relative to N123 leads to higher *f*_*d*_ specific SSLF values while *d*_*f*_ again is nearly unaffected in this parameter. Interestingly, the *d*_*f*_ specific SSPE values are affected by this setting resulting in an equivalent or slightly lower adjusted *R*^2^ compared to *f*_*d*_. Notably, the opposite is the case when decreasing the ancestral population size N12 relative to N123. In this case *d*_*f*_ shows higher SSPE values than *f*_*d*_ but in both cases, the adjusted *R*^2^ of both statistics are high and much greater than those for Patterson’s *D* as in other cases noted below.

**Table 1:** The effect of the distance to ancestral population

Direction of gene-flow	Distance to ancestral	*D*	*f*_*d*_	*d*_*f*_
	*t*_12_- *t*_123_- *t*_123*O*_			
*P*3→*P*2	1-2-3 (panel a)	0.39	0.80	0.81^a^
		1.41	0.09	0.00^b^
		0.48	0.19	0.25^c^
*P*2→*P*3	1-2-3 (panel b)	0.40	0.78	0.77 ^a^
		0.70	0.54	0.30^b^
		0.48	0.19	0.19^c^
*P*3→*P*2	1-1-3 (panel c)	0.58	0.77	0.70^a^
		0.12	0.40	0.04^b^
		0.60	0.17	0.35^c^
*P*2→*P*3	1-1-3 (panel d)	0.57	0.76	0.70^a^
		0.12	0.42	0.05^b^
		0.59	0.17	0.33^c^

This table refers to Fig. 2 and displays some supporting values

^a^the adjusted *R*^2^ ’goodness of fit’ (*higher is better*).

^b^SSLF ’sum of squares due to lack of fit’ divided by the sample size n=100 (*lower is better*).

^c^SSPE ’pure sum of squares error’ (*lower is better*).

### The effect of the time of gene-flow

**Fig. 3:**
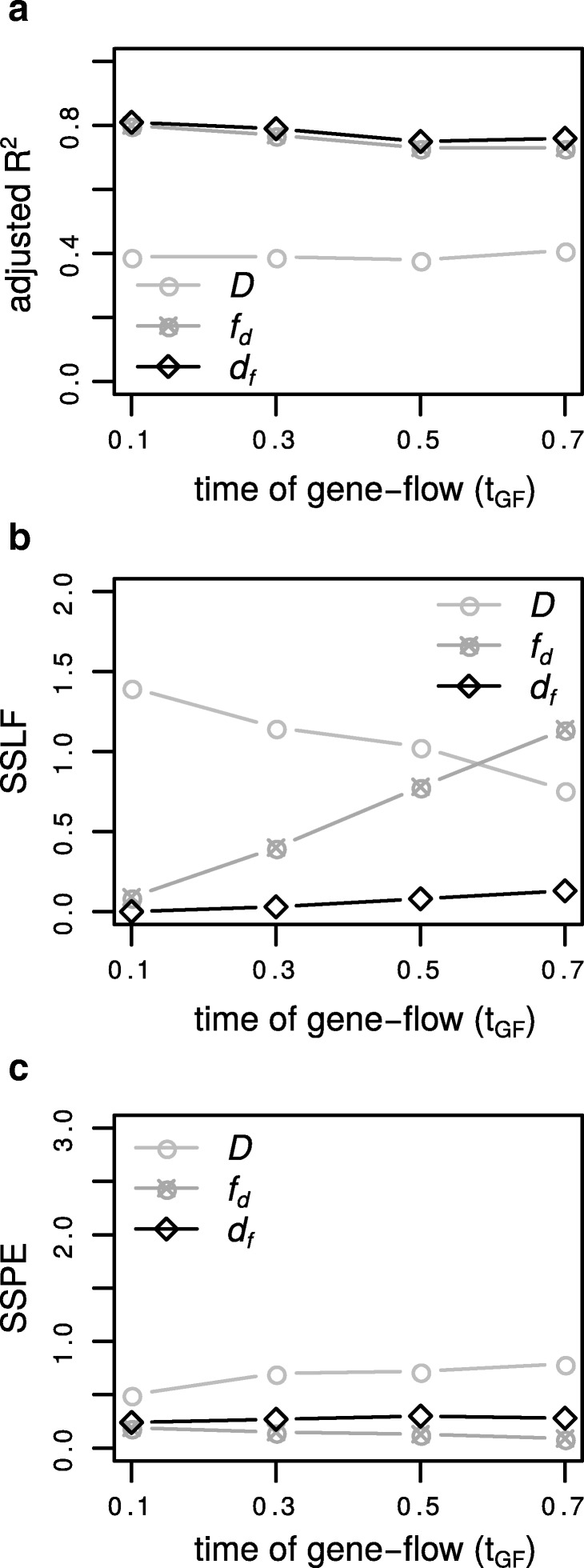
The effect of time of gene-flow. For *P*3→*P*2 introgression we varied the time of gene-flow (*t*_*GF*_=0.1, 0.3, 0.5, 0.7 ×4*N*) and calculated for each statistic (*D*, *f*_*d*_ and *d*_*f*_) **a** the adjusted *R*^2^ ’goodness of fit’. **b** SSLF ’sum of squares due to lack of fit’ divided by the sample size n=100. **c** SSPE ’pure sum of squares error’. A window size of 5kb and a recombination rate of r=0.01 was used. The background history is: P12= 1×4*N*, P123= 2×4*N* and P123O= 3×4*N* generations ago. The calls to the ms program can be found in the caption of Additional file 1: Table S1.3

One advantage of *d*_*f*_ compared to the other methods studied in this paper is that it is rarely affected by the time of gene-flow (Fig. 3). This is due to the fact that, unlike *f*_*d*_,*d*_*f*_ does not relate the signal of introgression to its maximum calculated from the present. When gene flow occurs in the distant past the denominator of *f*_*d*_ estimates increases leading to an underestimation of the fraction of introgression. The model fit shown by adjusted *R*^2^ of *d*_*f*_ is consistently higher than *f*_*d*_ (Fig. 3a), but more importantly, at the same time the SSLF values are almost unaffected by the time of gene-flow (Fig. 3b). Notably, we see the same effect when introgression is from *P*2→*P*3 (Additional file 1: Table S1.3) with *d*_*f*_ and *f*_*d*_ both showing higher adjusted *R*^2^ than Patterson’s *D* and a relatively low SSPE, yet, unlike the other direction, both show an increase in SSLF with time of gene-flow with *f*_*d*_ greater than *d*_*f*_.

### The effect of recombination and low variability

We found that all three methods *d*_*f*_, *f*_*d*_ and Patterson’s *D* become more accurate with increasing recombination rates. This is due to the increase of independent sites of a region analyzed. While *d*_*f*_ tends to have higher variances when the recombination rate is low it’s variance is comparable to *f*_*d*_ as soon as the recombination rate increases (see Additional file 1: Table S1.4). We also varied the scaled mutation rate (*θ*) to study the effect of low mutational genomic variability. Overall, *d*_*f*_ and *f*_*d*_ are only slightly affected by that parameter, whereas in comparison to the other methods *d*_*f*_ again showing the lowest SSLF values and with its goodness of fit (adjusted *R*^2^) slightly outperforming *f*_*d*_ (see Additional file 1: Table S1.5), while Patterson’s *D*, as in the other cases, performs more poorly than the other statistics in this comparison.

### The effect of window size and sample size

**Fig. 4:**
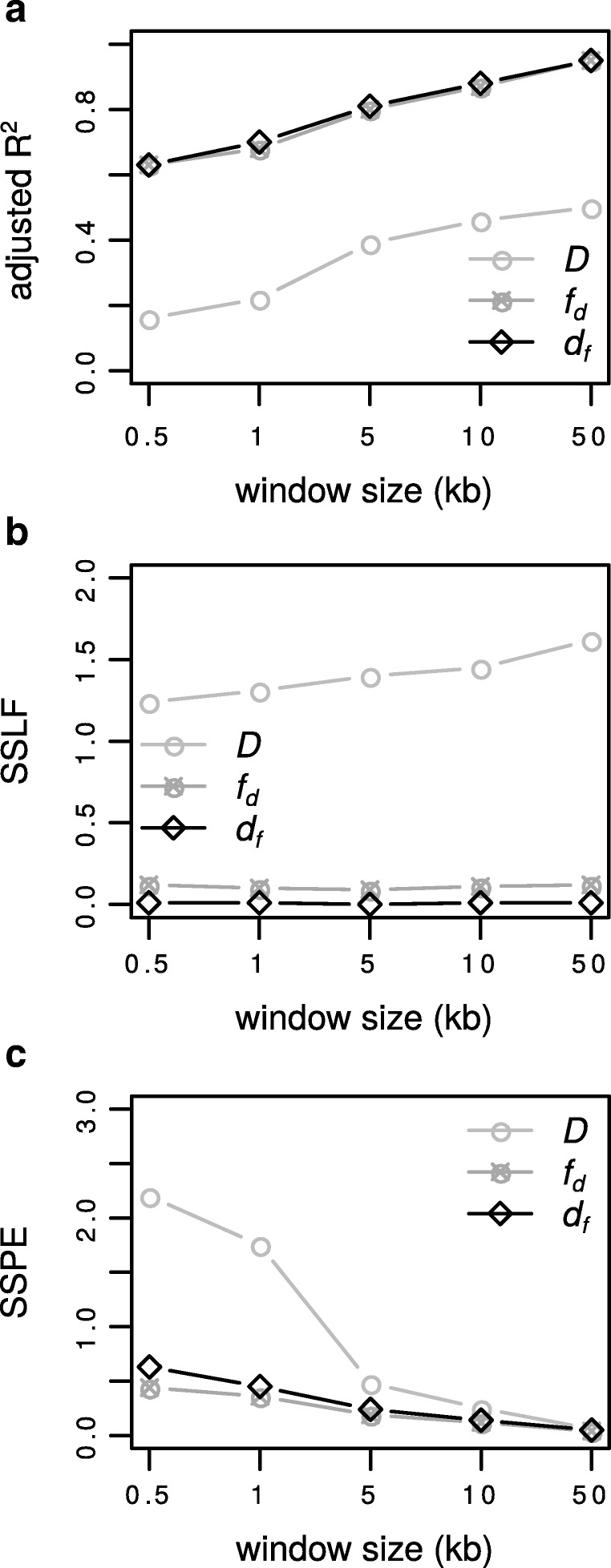
The effect of window size. For *P*3→*P*2 introgression we varied window sizes (0.5, 1, 5, 10, 50 kb) and calculated for each statistic (*D*, *f*_*d*_ and *d*_*f*_) **a** the adjusted *R*^2^ ’goodness of fit’. **b** SSLF ’sum of squares due to lack of fit’ divided by the sample size n=100. **c** SSPE ’pure sum of squares error’. The recombination rate is *r*=0.01. The background history is: P12= 1×4*N*, P123= 2×4*N* and P123O= 3×4*N* generations ago. Time of gene-flow is set to *t*_*GF*_=0.1×4*N* generations ago. The calls to the ms program can be found in the caption of Additional file 1: Table S1.6

As expected *d*_*f*_, *f*_*d*_ and Patterson’s *D* are more accurate with increasing genomic window size (varied from 0.5 kb to 50 kb, Fig. 4), however the latter performs much more poorly than the former statistics. As the window size increased both *d*_*f*_ and *f*_*d*_ show a nearly identical pattern of increasing goodness of fit (adjusted *R*^2^ from approximately 0.6 - 0.9 respectively) and corresponding near zero SSLF (with *d*_*f*_ slightly outperforming *f*_*d*_) and a decreasing SSPE, (with *f*_*d*_ slightly outperforming *d*_*f*_ at the two smallest window sizes; Fig. 4, Additional file 1: Table S1.6). Both *d*_*f*_ and *f*_*d*_ perform satisfactorily at all windows sizes tested. In contrast, the Patterson’s *D* shows a poor goodness of fit, a much larger SSLF and for the two smallest window sizes, a much larger SSPE. Note sample size had very little effect overall (Additional file 1: Table S1.7).

### On the ability to detect introgression

In this simulation scenario *d*_*f*_ and the *f*_*d*_ estimate show nearly the same utility (higher is better) for the fraction of introgression and distance to ancestral population (Additional file 1: Section S2); but both greatly outperform the Patterson’s *D* statistic especially for smaller genomic regions. We also included the recently published *RNDmin* [29] method in this latter analysis; this alternative only gives good results when the signal of introgression is very strong (Additional file 1: Section S2). In addition, unlike *f*_*d*_, *d*_*f*_ is able to quantify the proportion of admixture symmetrically (*P*3⇔*P*2 and *P*3⇔*P*1) thus simplifying the analysis of real genomic data on a 4-taxon system.

### Application

**Fig. 5:**
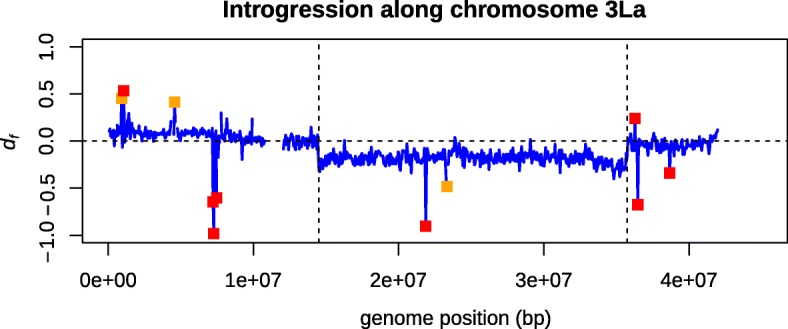
Anopheles gambiae 3La inversion. Confirming introgression on the 3L arm of the malaria vector *Anopheles gambiae* (Fontaine et al. 2015, Fig. 4). The area between the vertical dashed lines delineate the introgressed chromosomal inversion. We used the R-package PopGenome to scan the chromosome with 50kb consecutive windows and plotted the *d*_*f*_ values along the chromosome (*Laplace smoothed*). Orange boxes indicate outlier windows below a significance level of 0.05 and red boxes show outlier windows on the basis of a 0.01 significance level. The p-values were corrected for multiple testing by the Benjamini-Hochberg method

Figure 5 shows *d*_*f*_ for 50kb consecutive windows on the 3L arm of malaria vectors in the *Anopheles gambiae* species complex confirming the recently detected region of introgression found in an inversion [17]. Outliers detected both inside and outside the inversion are shown in Table 2.

**Table 2:** Significant outliers detected on the *Anopheles gambiae* 3La chromosome

Mb (start)	Mb (end)	*d*_*f*_	*Z*
0.90	0.95	0.45	2.05^*^
1.05	1.10	0.53	2.41^**^
4.55	4.60	0.41	1.87^*^
7.20	7.25	-0.65	-2.92^**^
7.25	7.30	-0.98	-4.45^**^
7.45	7.50	-0.60	-2.73^**^
21.85	21.90	-0.90	-5.91^**^
23.30	23.35	-0.48	-2.45^*^
26.25	26.30	0.24	2.28^**^
36.45	36.50	-0.68	-6.42^**^
38.65	38.70	-34	-3.22^**^

^*^0.05 significance level

^**^0.01 significance level

Overall, we found 9 significant outliers outside the inversion and two outliers within the inversion based on a 0.05 significance level (see Fig. 5). This further reduces to 7 significant outliers outside the inversion and one remaining outlier within the inversion when tested against a 0.01 significance level (see Table 2).

These analyses were all performed within the R package PopGenome [28] and can be easily reproduced with the code given in the Additional file 1: Section S3.

## Discussion

In the last 8 years there has been an explosion of population genomic methods to detect introgression. The Patterson’s *D* method, based on patterns of alleles in a four-taxon comparison, has been widely applied to a variety of problems that differ from those for which it was originally developed. This statistic can be used to assess whether or not introgression is occurring at the whole genome scale, however, Patterson’s *D* is best not applied to smaller genomic regions or gene-scans as noted by Martin et al. 2015.

The distance based approach proposed here has the following strengths: First, the approach is based on characterizing changes in genetic distances that are a natural consequence of introgression. Second, distance measured by *d*_*xy*_ allows direct comparisons of quantities that are easily interpreted. Third, the distance fraction, *d*_*f*_, accurately predicts the fraction of introgression over a wide-range of simulation parameters. Furthermore, the *d*_*f*_ statistic is symmetric (like Patterson’s *D*) which makes it easy to implement and interpret. Yet, *d*_*f*_ outperforms Patterson’s *D* in all cases (the latter shows a strong positive bias) and *d*_*f*_ also outperforms or is equivalent to *f*_*d*_ in nearly all cases judged by the goodness of fit and the sum of squares due to lack of fit. Furthermore, unlike *f*_*d*_, *d*_*f*_ does not vary strongly with the time of gene-flow. This latter strength comes from incorporating the shared genetic distance to taxon 3 (P3) into the denominator, serving to scale *d*_*f*_ relative to *d*_*xy*_ values between the three species in the comparisons. Ultimately this makes the statistic *less* subject to extreme values due to low SNP diversity (low genetic distances), as evidence by lower values than other statistics in our examples.

**Fig. 6:**
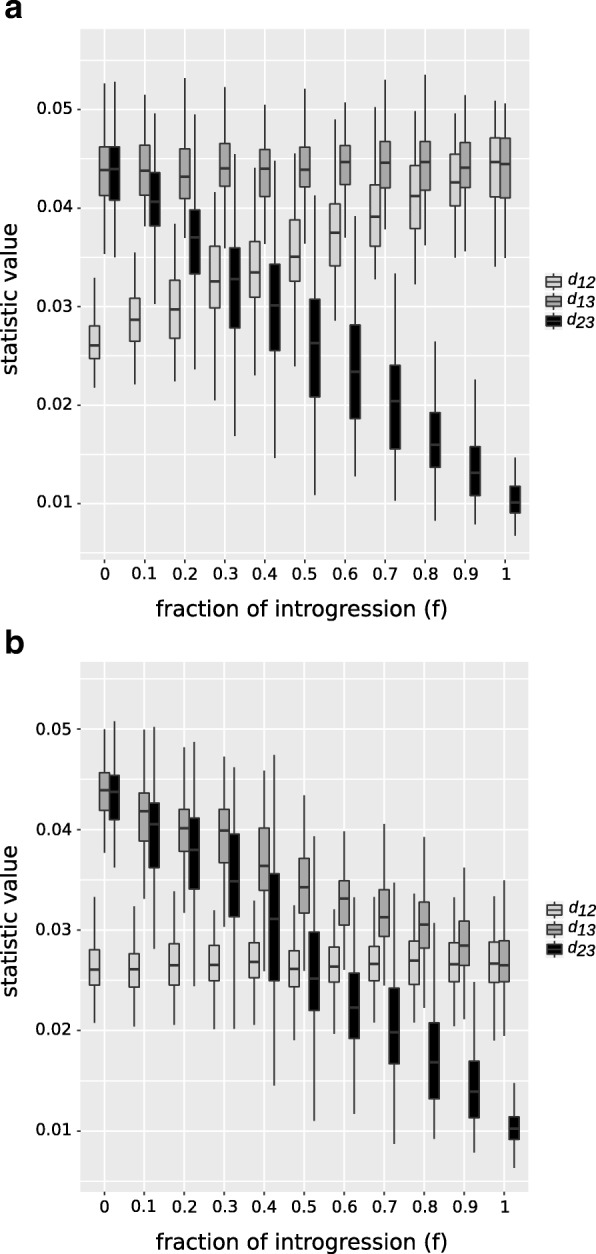
The effect of introgression on pairwise distances. The effect of the fraction of introgression on the average pairwise distance measurements *d*_12_, *d*_13_ and *d*_23_. **a** The effect is shown for *P*3→*P*2 introgression. **b** Shows the effect in case of *P*2→*P*3 introgression. The background history is: P12= 1×4*N*, P123= 2×4*N* and P123O= 3×4*N* generations ago. Time of gene-flow is set to *t*_*GF*_=0.1×4*N* generations ago. The calls to the ms program can be found in the example from the methods section

There are several areas where further improvements could be made. Although the distance based derivation of all three statistics is sound, and *d*_*f*_ is empirically supported by simulation, further mathematical analysis for this general class of distance estimators is desired. Like other statistics under consideration in this paper, *d*_*f*_ depends on resolved species tree with a particular configuration of two closely related species, a third species and an outgroup, and therefore it is not directly applicable to other scenarios. In addition, both the *f*_*d*_ and *d*_*f*_ perform less accurately when measuring the proportion of admixture when the gene-flow occurs from P2 to P3. On the other hand, our simulations show (Fig. 6) the asymmetrical effect of gene-flow direction on genetic distance: gene-flow from P3 to P2 does not affect the distance between taxon 1 & 3 (*d*_13_), however, the opposite it true when introgression from P2 to P3 occurs, the distance between taxon 1 & 2 (*d*_12_) is not affected. This suggests comparisons of *d*_*xy*_ within given genomic regions may contain signal to infer the direction of introgression and therefore more accurately measure the proportion of admixture.

Overall, the distance based interpretation of introgression statistics suggests a general framework for estimation of the fraction of introgression on a known tree and may be extended in a few complementary directions including the use of model based approaches to aid in outlier identification and potentially model selection. The distance based framework introduced here may lead to other further improvements by measuring how genetic distance changes between different taxa as a function of hybridization across different parts of the genome.

## Conclusion

Here we present both a simplified distance based interpretation for Patterson’s *D* and Martin et al.’s *f*_*d*_ and a new distance based statistic *d*_*f*_ that avoids the pitfalls of Patterson’s *D* when applied to small genomic regions and is more accurate and less prone to vary with variation in the time of gene flow than *f*_*d*_. We propose *d*_*f*_ as an estimate of introgression which can be used to simultaneously detect and quantify introgression. We implement *d*_*f*_ (as well as the other four-taxon statistics, *f*_*d*_, and the original Patterson’s *D*) in the powerful R-package, PopGenome [28], now updated to easily calculate these statistics for individual loci to entire genomes.

## Additional file

Additional file 1 **Section S1** On the Accuracy to Measure the Real Fraction of Introgression. **Section S2** Detecting Introgression from Whole Genome Data. **Section S3** PopGenome Usage. (PDF 275 kb)

## Abbreviations

FDR: False discovery rate (Benjamini-Hochberg method)
*R*^2^: Adjusted *R*^2^ (a measure of ‘goodness of fit’)
SSLF: Sum of squares due to lack of fit (a measure of bias)
SSPE: Pure sum of squares error
SNPs: Single-nucleotide polymorphisms
