# Supplementary material for: Estimates of introgression as a function of pairwise distances
Source: BMC Bioinformatics. 2019 Apr 23;20:207. doi: 10.1186/s12859-019-2747-z (PMC6480520; doi:10.1186/s12859-019-2747-z)
Supplement: Supplementary file 1 — Section S1 On the Accuracy to Measure the Real Fraction of Introgression. Section S2 Detecting Introgression from Whole Genome Data. Section S3 PopGenome Usage. (PDF 275 kb) [file 12859_2019_2747_MOESM1_ESM.pdf]

Estimates of Introgression as a Function of Pairwise Distances

SUPPLEMENTAL INFORMATION

S1 On the Accuracy to Measure the Real Fraction of Introgression **2**
Distance of ancestral population 2
Supplementary Table S1.1. Distance to ancestral population. 2
Ancestral population sizes 4
Supplementary Table S1.2. Ancestral population sizes. 4
Time of gene-flow 5
Supplementary Table S1.3. Effect of the time of gene-flow. 5
Recombination 6
Supplementary Table S1.4. Recombination. 6
The effect of low variability 7
Supplementary Table S1.5. The effect of low variability. 7
The effect of window size 8
Supplementary Table S1.6. The effect of window size. 8
The effect of sample size 9
Supplementary Table S1.7. The effect of sample size. 9
S2 Detecting Introgression from Whole Genome Data **9**
Fraction of introgression 10
Supplementary Table S2.1. Fraction of introgression. 10
Distance of ancestral population 10
Supplementary Table S2.2. Distance of ancestral population. 11
S3 PopGenome Usage **11**

S1 On the Accuracy to Measure the Real Fraction of Introgression

Distance of ancestral population

Starting with the following topology (((P1, P2), P3), O) we simulate varying depths to common ancestors of P1 & P2, and at the root (P123O), where recombination rate is fixed with 4*Nr* =50 (*r*=0.01) for each direction of gene flow.

Supplementary Table S1.1. Distance to ancestral population.

Direction of gene-flow	Distance to ancestral population (t_{12}-t_{123}-t_{123O})	*D*	f_d	d_f	
P3→P2	0.3-1-3	0.4388	0.7662	0.7739	a
		0.7060	0.2893	0.1884	b
		0.5175	0.1667	0.2210	c
P3→P2	0.5-1-3	0.4916	0.7622	0.7837	a
		0.4895	0.3283	0.1278	b
		0.5619	0.1676	0.2180	c
P3→P2	0.7-1-3	0.5158	0.7675	0.7698	a
		0.2928	0.3667	0.0733	b
		0.6544	0.1695	0.2487	c
P3→P2	1-1-3	0.5846	0.7733	0.7014	a
		0.1177	0.4003	0.0380	b
		0.6033	0.1667	0.3540	c
P3→P2	0.5-2-3	0.3314	0.7901	0.8064	a
		1.6221	0.0814	0.0798	b
		0.6026	0.2005	0.2252	c
P3→P2	0.5-3-3	0.2704	0.8048	0.8077	a
		1.9736	0.0299	0.0884	b
		0.6129	0.2087	0.2255	c
P3→P2	1-2-3	0.3905	0.7978	0.8115	a
		1.4075	0.0928	0.0048	b
		0.4839	0.1931	0.2361	c
P3→P2	1-3-3	0.3267	0.8142	0.8212	a
		1.7027	0.0343	0.0269	b
		0.6096	0.2039	0.2297	c

P3→P2	2-2-3	0.5858	0.8115	0.7254	a
		0.4688	0.1920	0.1339	b
		0.4950	0.1761	0.3274	c
P2→P3	0.3-1-3	0.1593	0.4724	0.4320	a
		0.8882	1.8376	2.2058	b
		0.7641	0.1412	0.1072	c
P2→P3	0.5-1-3	0.3660	0.6895	0.6465	a
		0.2165	1.0807	1.0442	b
		0.7532	0.1314	0.1662	c
P2→P3	0.7-1-3	0.4925	0.7283	0.7001	a
		0.1415	0.6811	0.3466	b
		0.6317	0.1551	0.2418	c
P2→P3	1-1-3	0.5745	0.7569	0.7040	a
		0.1211	0.4169	0.0473	b
		0.5876	0.1742	0.3340	c
P2→P3	0.5-2-3	0.2525	0.6755	0.6183	a
		0.3321	1.2255	1.4610	b
		0.8886	0.1285	0.1333	c
P2→P3	0.5-3-3	0.2361	0.6709	0.5852	a
		0.3760	1.3214	1.6903	b
		0.9414	0.1249	0.1160	c
P2→P3	1-2-3	0.3952	0.7778	0.7691	a
		0.6901	0.5376	0.3026	b
		0.4839	0.1931	0.1895	c
P2→P3	1-3-3	0.3767	0.7589	0.7226	a
		0.8946	0.6210	0.6119	b
		0.6854	0.1570	0.1924	c
P2→P3	2-2-3	0.5628	0.8020	0.7188	a
		0.4822	0.1948	0.1419	b
		0.5399	0.1839	0.3309	c

For each direction of gene flow and distance to ancestral populations (see above) we calculated for each statistic (D, f_d and d_f) [a] the adjusted R^2 'goodness of fit'. [b] *SSLF* 'sum of squares due to lack of fit' divided by the sample size n=100. [c] SSPE 'pure sum of squares error'. The time of gene-flow was a constant at 0.1×4*N*, the scaled recombination rate is 4*Nr*=50 (*r*=0.01), and the calls to *ms* are as follows:

P3→P2: ms 32 1 -I 4 8 8 8 8 -ej t_{12} *2 1 -ej* t_{123} *3 1 -ej* t_{123O} *4 1 -es 0.1 2* ***Fraction*** *-ej 0.1 5 3 -r 50 5000*

P2→P3: ms 32 1 -I 4 8 8 8 8 -ej t_{12} 2 1 -ej t_{123} 3 1 -ej t_{123O} 4 1 -es 0.1 3 ***Fraction*** *-ej 0.1 5 2 -r 50 5000*

Ancestral population sizes

We varied ancestral population sizes at the nodes P12 and P123 and simulated the impact on the (D, f_d and d_f) statistics (see supplementary table S1.2 below).

Supplementary Table S1.2. Ancestral population sizes.

Direction of gene-flow	Ancestral population size N12 vs N123	D	f_d	d_f	
P3→P2	2-1	0.4857	0.8177	0.8237	a
		0.9736	0.1292	0.0036	b
		0.4743	0.1743	0.2197	c
P3→P2	10-1	0.5790	0.8000	0.7677	a
		0.6137	0.1867	0.0649	b
		0.4147	0.1841	0.2693	c
P3→P2	10-2	0.6208	0.7957	0.7778	a
		0.4066	0.1200	0.0147	b
		0.4288	0.2109	0.2804	c
P3→P2	1-2	0.4133	0.8275	0.8342	a
		1.1476	0.0668	0.0304	b
		0.6070	0.1809	0.2065	c
P3→P2	1-10	0.4606	0.8031	0.8062	a
		1.0477	0.0084	0.1009	b
		0.4794	0.2411	0.2165	c
P3→P2	2-10	0.5955	0.7950	0.8022	a
		0.5244	0.0095	0.0474	b
		0.4276	0.2555	0.2420	c

For different ancestral population sizes (multiples of 1, 2 and 10×4*N*) at nodes P12 and P123 we calculated for each statistic (D, f_d and d_f) and present [a] the adjusted R^2 'goodness of fit'. [b] *SSLF* 'sum of squares due to lack of fit' divided by the sample size n=100. [c] SSPE 'pure sum of squares error'. The time of gene-flow (t_{GF}) is 0.1×4*N*, scaled recombination rate is 4*Nr*=50 (*r*=0.01), and background history is: P12=1×4*N*, P123=2×4*N* and P123O=3×4*N*, and the calls to *ms* are:

*P3→P2: ms 32 1 -I 4 8 8 8 8 -ej 1 2 1 -en 1.01 1 **N12** -ej 2 3 1 -en 2.01 1 **N123** -ej 3 4 1 -es 0.1 2 **Fraction** -ej 0.1 5 3 -r 50 5000*

Time of gene-flow

Supplementary Table S1.3. Effect of the time of gene-flow.

Direction of gene-flow	Time of Gene-flow (t_{GF})	D	f_d	d_f	
P3→P2	0.1	0.3905	0.7978	0.8115	a
		1.4075	0.0928	0.0048	b
		0.4839	0.1931	0.2361	c
P3→P2	0.3	0.3918	0.7681	0.7870	a
		1.1462	0.4039	0.0255	b
		0.6805	0.1530	0.2659	c
P3→P2	0.5	0.3805	0.7291	0.7525	a
		1.0277	0.7815	0.0782	b
		0.7240	0.1250	0.2924	c
P3→P2	0.7	0.4084	0.7308	0.7620	a
		0.7601	1.1447	0.1341	b
		0.7850	0.0895	0.2751	c
P2→P3	0.1	0.3952	0.7778	0.7691	a
		0.6901	0.5376	0.3026	b
		0.4839	0.1931	0.1895	c
P2→P3	0.3	0.3702	0.6938	0.7003	a
		0.4257	1.2463	0.5155	b
		0.8134	0.1078	0.2379	c
P2→P3	0.5	0.3069	0.5779	0.5936	a
		0.2800	2.0306	0.9803	b
		0.9632	0.0690	0.2356	c
P2→P3	0.7	0.1639	0.4283	0.4617	a
		0.4376	2.7323	1.7089	b
		1.3510	0.0432	0.1957	c

For each direction of introgression we varied the time of gene-flow (0.1, 0.3, 0.5, 0.7 × 4N) and calculated for each statistic (D, f_d and d_f) [a] the adjusted R^2 'goodness of fit'. [b] SSLF 'sum of squares due to lack of fit' divided by the sample size n=100. [c] SSPE 'pure sum of

squares error'. Scaled recombination rate is 4*Nr*=50 (*r*=0.01). The background history is: P12=1×4*N*, P123=2×4*N* and P123O=3×4*N* generations ago. The calls to *ms* are:

P3→P2: ms 32 1 -I 4 8 8 8 8 -ej 1 2 1 -ej 2 3 1 -ej 3 4 1 -es $\mathbf{t_{GF}}$ *2* ***Fraction*** *-ej* $\mathbf{t_{GF}}$ *5 3 -r 50 5000*

Recombination

To test the impact of recombination on these statistics we varied the recombination rates from (*r* = 0 - .08). With increasing recombination rates the accuracy to measure the real fraction of introgression increases for f_d and d_f while the Patterson's *D* is rarely affected by varying this parameter.

Supplementary Table S1.4. Recombination.

Direction of gene-flow	Recombination rate *r*	*D*	f_d	d_f	
P3→P2	0/5000	0.3307	0.6335	0.6062	a
		0.7697	0.1066	0.0139	b
		1.4885	0.4485	0.6535	c
P3→P2	50/5000	0.3905	0.7978	0.8115	a
		1.4075	0.0928	0.0048	b
		0.4839	0.1931	0.2361	c
P3→P2	100/5000	0.4072	0.8676	0.8758	a
		1.4905	0.1114	0.0080	b
		0.3725	0.1155	0.1467	c
P3→P2	200/5000	0.4046	0.8986	0.9020	a
		1.5117	0.1163	0.0121	b
		0.3639	0.0864	0.1096	c
P3→P2	300/5000	0.4069	0.9249	0.9257	a
		1.5641	0.1209	0.0150	b
		0.3191	0.0625	0.0820	c
P3→P2	400/5000	0.4041	0.9383	0.9360	a
		1.5778	0.1223	0.0169	b
		0.3186	0.0510	0.0691	c

For recombination rates varying from (0 to .08) we calculated for each statistic (*D*, f_d and d_f) [a] the adjusted R^2 'goodness of fit'. [b] *SSLF* 'sum of squares due to lack of fit' divided by the sample size n=100 . [c] SSPE 'pure sum of squares error'. The time of gene-flow (t_{GF})

was a constant at 0.1×4*N*, background history is P12=1×4*N*, P123=2×4*N* and P1234=3×4*N*, and the calls to *ms* are as follows:

P3→P2: ms 32 1 -I 4 8 8 8 8 -ej 1 2 1 -ej 2 3 1 -ej 3 4 1 -es 0.1 2 ***Fraction*** *-ej 0.1 5 3 -r* ***r*** **× 5000** *5000*

The effect of low variability

We varied the nucleotide diversity θ to test the effect of low variability on the statistics *D*, f_d and d_f.

Supplementary Table S1.5. The effect of low variability.

Direction of gene-flow	Variability theta (θ)	*D*	f_d	d_f	
P3→P2	3	0.3960	0.8153	0.8197	a
		1.3084	0.1108	0.0130	b
		0.5743	0.1772	0.2252	c
P3→P2	5	0.4015	0.8083	0.8205	a
		1.3239	0.1218	0.0192	b
		0.5362	0.1832	0.2206	c
P3→P2	25	0.4035	0.7991	0.8102	a
		1.2020	0.1310	0.0229	b
		0.6193	0.1923	0.2379	c
P3→P2	50	0.4092	0.8068	0.8217	a
		1.3574	0.1009	0.0064	b
		0.4953	0.1866	0.2277	c

For unidirectional gene-flow P3→P2 for 5kb sequences, we varied *θ* from 3 - 50 and calculated for each statistic (*D*, f_d and d_f) and present [a] the adjusted R^2 'goodness of fit'. [b] *SSLF* 'sum of squares due to lack of fit' divided by the sample size n=100. [c] SSPE 'pure sum of squares error'. The time of gene-flow (t_{GF}) was a constant at 0.1×4*N*, the scaled recombination rate is 4*Nr*=50 (*r*=0.01) and background history is P12=1×4*N*, P123=2×4*N* and P123O=3×4*N*. The calls to *ms* are:

P3→P2: ms 32 1 -I 4 8 8 8 8 -ej 1 2 1 -ej 2 3 1 -ej 3 4 1 -es 0.1 2 ***Fraction*** *-ej 0.1 5 3 -r 50 5000 -t* ***theta***

The effect of window size

We varied the size of the window (kb) to test this effect on the statistics *D*, f_d and d_f.

Supplementary Table S1.6. The effect of window size.

Direction of gene-flow	Window size (kb)	*D*	f_d	d_f	
P3→P2	0.5	0.16	0.63	0.63	a
		1.24	0.12	0.01	b
		2.20	0.44	0.63	c
P3→P2	1	0.22	0.68	0.70	a
		1.31	0.10	0.01	b
		1.75	0.36	0.45	c
P3→P2	5	0.39	0.80	0.81	a
		1.40	0.09	0	b
		0.48	0.19	0.24	c
P3→P2	10	0.46	0.87	0.88	a
		1.45	0.11	0.01	b
		0.25	0.12	0.14	c
P3→P2	50	0.50	0.95	0.95	a
		1.62	0.12	0.01	b
		0.06	0.04	0.05	c

For unidirectional gene-flow P3→P2, we varied the window size and calculated for each statistic (*D*, f_d and d_f) and present [a] the adjusted R^2 'goodness of fit'. [b] *SSLF* 'sum of squares due to lack of fit' divided by the sample size n=100. [c] SSPE 'pure sum of squares error'. The time of gene-flow (t_{GF}) was at 0.1×4*N*, the scaled recombination rate is *r*=0.01 and background history is P12=1×4*N*, P123=2×4*N* and P123O=3×4*N*. The calls to *ms* are: *P3→P2: ms 32 1 -I 4 8 8 8 8 -ej 1 2 1 -ej 2 3 1 -ej 3 4 1 -es 0.1 2* ***Fraction*** *-ej 0.1 5 3 -r* ***kb×10 kb×1000***

The effect of sample size

We varied the sample size (*s*) to test this effect on the statistics *D*, f_d and d_f.

Supplementary Table S1.7. The effect of sample size.

Direction of gene-flow	Sample size *s*	*D*	f_d	d_f

P3→P2	8	0.3905	0.7978	0.8115	[a]
		1.4075	0.0928	0.0048	[b]
		0.4839	0.1931	0.2361	[c]
P3→P2	20	0.3794	0.8119	0.8227	[a]
		1.4429	0.0695	0.0112	[b]
		0.4880	0.1911	0.2142	[c]
P3→P2	50	0.3831	0.8076	0.8238	[a]
		1.4469	0.053	0.0102	[b]
		0.4996	0.1969	0.2115	[c]
P3→P2	100	0.3803	0.8362	0.8472	[a]
		1.4267	0.0561	0.0116	[b]
		0.4931	0.1673	0.1840	[c]

For unidirectional gene-flow P3→P2, we varied the sample size and calculated for each statistic (*D*, f_d and d_f) and present [a] the adjusted R^2 'goodness of fit'. [b] *SSLF* 'sum of squares due to lack of fit' divided by the sample size n=100. [c] SSPE 'pure sum of squares error'. The time of gene-flow (t_{GF}) was a constant at 0.1×4*N*, the scaled recombination rate is 4*Nr*=50 (*r*=0.01) and background history is P12=1×4*N*, P123=2×4*N* and P123O=3×4*N*. The calls to *ms* are:

P3→P2: ms 4×s 1 -I 4 ***s s s s*** *-ej 1 2 1 -ej 2 3 1 -ej 3 4 1 -es 0.1 2* ***Fraction*** *-ej 0.1 5 3 -r 50 5000*

S2 Detecting Introgression from Whole Genome Data

To test the performance of the various statistics (*D*, f_d, *RNDmin*, d_f) to distinguish neutral models from models with varying levels of introgression or varying distances to ancestral populations, we performed two simulations on 1kb windows. For each statistic of interest we present the area under the curve (AUC) values. All simulations start with 10,000 loci under the neutral scenario (*f=0*) and 1,000 loci with subject to introgression. The Recombination rate is fixed at *r=0.01*.

Fraction of introgression

To test the impact of varying the fraction of introgression we simulated the fraction of introgression for the 'alternative model' from *f*=0.1 to *f*=1 and compared this to the neutral scenario where the fraction of introgression is zero (*f=0*). See below (supplementary Table S2.1).

Supplementary Table S2.1. Fraction of introgression.

Direction of gene-flow	Fraction of introgression *f*	*D*	f_d	*RNDmin*	d_f
P3→P2	0.1	0.6252	0.7128	0.5579	0.7065
P3→P2	0.2	0.6846	0.8426	0.6043	0.8330
P3→P2	0.3	0.7163	0.9221	0.6479	0.9139
P3→P2	0.4	0.7293	0.9541	0.7196	0.9478
P3→P2	0.5	0.7380	0.9810	0.7588	0.9753
P3→P2	0.6	0.7466	0.9922	0.8246	0.9890
P3→P2	0.7	0.7585	0.9979	0.8510	0.9961
P3→P2	0.8	0.7607	0.9988	0.9216	0.9980
P3→P2	0.9	0.7748	1	0.9659	0.9996
P3→P2	1	0.7871	1	1	0.9998

The effect of varying fractions of introgression on the model utility in the ROC analysis as indicated for values of AUC. The background history (coalescent times) is: *P12=1×4N*, *P123=2×4N* and *P123O=3×4N* generations ago. The time of gene-flow (t_{GF}) was set to 0.1×4*N* generations ago and the recombination rate is *r*=0.01. The calls to *ms* are:

Neutral model: ms 32 1 -I 4 8 8 8 8 -ej 1 2 1 -ej 2 3 1 -ej 3 4 1 -r 10 1000

Alternative model: P3→P2: ms 32 1 -I 4 8 8 8 8 -ej 1 2 1 -ej 2 3 1 -ej 3 4 1 -es 0.1 2 ***(1-f)*** *-ej 0.1 5 3 -r 10 1000*

Distance of ancestral population

Finally, by varying the distance to ancestral populations we tested the impact of a low amount of introgression on the various statistics.

Supplementary Table S2.2. Distance of ancestral population.

Direction of gene-flow	Distance to ancestral population (t_{12}-t_{123}-t_{123O})	*D*	f_d	*RNDmin*	d_f
P3→P2	0.3-1-3	0.6366	0.6860	0.5308	0.6782
P3→P2	1-1-3	0.5884	0.6077	0.5107	0.5969
P3→P2	0.5-2-3	0.6314	0.7292	0.5489	0.7272
P3→P2	0.5-3-3	0.6454	0.7493	0.5498	0.7488
P3→P2	1-2-3	0.6252	0.7128	0.5482	0.7065

P3→P2	1-3-3	0.6465	0.7604	0.5690	0.7575
P3→P2	2-2-3	0.6016	0.6406	0.5312	0.6220
P3→P2	1.5-2-3	0.6065	0.6680	0.5526	0.6573

10.000 loci under the neutral scenario (f=0). Fraction of introgression for the 'alternative model' simulations is f=0.1 (1,000 loci). Recombination rate is $r=0.01$. Time of gene-flow (t_{GF}) is 0.1×4N generations ago. The calls to *ms* are:

Neutral model:

ms 32 1 -I 4 8 8 8 8 -ej $\mathbf{t_{12}}$ *2 1 -ej* $\mathbf{t_{123}}$ *3 1 -ej* $\mathbf{t_{1234}}$ *4 1 -r 10 1000*

Alternative model:

P3→P2: ms 32 1 -I 4 8 8 8 8 -ej $\mathbf{t_{12}}$ *2 1 -ej* $\mathbf{t_{123}}$ *3 1 -ej* $\mathbf{t_{1234}}$ *4 1 -es 0.1 2* ***0.9*** *-ej 0.1 5 3 -r 10 1000*

S3 PopGenome Usage

```
# Install and load supporting packages from CRAN/GitHub within R
install.packages("dplyr")
install.packages("devtools")

library(dplyr)
library(devtools)

#Install PopGenome
#install.packages("PopGenome")

# Install the PopGenome package from github
install_github("pievos101/PopGenome")

# Load the PopGenome package
library(PopGenome)

###
Read the data (Fontaine, M. C., Pease, J. B., Steele, A., Waterhouse, R.
M., Neafsey, D. E., Sharakhov, I. V., et al. (2015). Mosquito genomics.
```

```
Extensive introgression in a malaria vector species complex revealed by
phylogenomics. Science, 347(6217), 1258524-1258524.
http://doi.org/10.1126/science.1258524)
###

genome =
readVCF("AGC_refHC_bialSNP_AC2_2DPGQ.3L_V2.CHRcode2.DRYAD.vcf.gz",
10000,"4",1,45000000, include.unknown=TRUE)

# Define the populations
Aquad = c("SRS408143", "SRS408145", "SRS408151", "SRS408155",
"SRS408966", "SRS408969", "SRS408972", "SRS408973", "SRS408983",
"SRS420578")
Amela = c("SRS408142", "SRS408185", "SRS408994")
Ameru = c("SRS408186", "SRS408187", "SRS408967", "SRS408974",
"SRS408992", "SRS410266","SRS410284", "SRS410286", "SRS410290",
"SRS420577")

# Define the outgroup
Chris = c("CHRISTYI")

# Set the populations
genome = set.populations(genome, list(Aquad,Amela,Ameru),diploid=TRUE)

# Set the outgroup
genome  = set.outgroup(genome, Chris, diploid=TRUE)

# Perform the weighted jackknife for the inverted region
inversionStart=1.45e07
inversionEnd=3.575e07

# Split the data into 50kb windows within the inversion
```

```
slide =
sliding.window.transform(genome,jump=50000,width=50000,start.pos=inversio
nStart, end.pos=inversionEnd, type=2)

# Perform the introgression statistics
slide  = introgression.stats(slide, l.smooth=TRUE)

# weighted jackknife
slide = weighted.jackknife(slide, per.region=FALSE)

# show results
slide@D.z
slide@D.pval
slide@df.z
slide@df.pval

# standard error
slide@df.SE
slide@D.SE

# Test for df outlier within the the inversion

# Calculate Z-values
meandf = mean(slide@df, na.rm=TRUE)
Z  = (as.vector(slide@df)-meandf)/as.vector(slide@df.SE)

# Calculate P-values
P = 2*(pnorm(-abs(Z)))

# BH correct the P-values
adjP = p.adjust(1-P, "BH")

# get significant loci
```

```
out.ids = which((1-adjP)<0.05)
out.ids2 = which((1-adjP)<0.01)

# Get the genomic positions
genome.pos = sapply(slide@region.names,function(x){
  split = strsplit(x," ")[[1]][c(1,3)]
  val = mean(as.numeric(split))
  return(val)
})

#save inversion results
outINV   = out.ids
outINV2  = out.ids2
slideINV = slide
genome.pos.INV = genome.pos
outINV_names  = slide@region.names[out.ids]
outINV2_names = slide@region.names[out.ids2]
Z_INV = Z

# Test for df outliers from the beginning of the chromosome 3L to the
start of the inversion

# First, split the data into 50kb windows outside the inversion
slide =
sliding.window.transform(genome,jump=50000,width=50000,start.pos=1,
end.pos=inversionStart, type=2)

# Perform the introgression statistics
slide  = introgression.stats(slide, l.smooth=TRUE)



# weighted jackknife
slide = weighted.jackknife(slide, per.region=FALSE)
```

```
# Calculate Z-values (Ho == 0)
Z  = (as.vector(slide@df)-0)/as.vector(slide@df.SE)

# Calculate P-values
P = 2*(pnorm(-abs(Z)))

# BH correct the P-values
adjP = p.adjust(1-P, "BH")

# get significant loci
out.ids = which((1-adjP)<0.05)
out.ids2 = which((1-adjP)<0.01)

slide@region.names[out.ids]

# get genomic positions
genome.pos = sapply(slide@region.names,function(x){
  split = strsplit(x," ")[[1]][c(1,3)]
  val = mean(as.numeric(split))
  return(val)
})

# save the results
outLeft = out.ids
outLeft2 = out.ids2
slideOUTleft = slide
genome.pos.Left = genome.pos
outLeft_names  = slide@region.names[out.ids]
outLeft2_names = slide@region.names[out.ids2]
Z_Left = Z
```

```
# Test for df outlier outside the the inversion to the end of chromosome
3L

# First, split the data into 50kb windows outside the inversion
slide =
sliding.window.transform(genome,jump=50000,width=50000,start.pos=inversio
nEnd, end.pos=45000000, type=2)

# Perform the introgression statistics
slide  = introgression.stats(slide, l.smooth=TRUE)

# weighted jackknife
slide = weighted.jackknife(slide, per.region=FALSE)

# Calculate Z-values
Z  = (as.vector(slide@df)-0)/as.vector(slide@df.SE)

# Calculate P-values
P = 2*(pnorm(-abs(Z)))

# BH correct the P-values
adjP = p.adjust(1-P, "BH")

# get significant loci
out.ids = which((1-adjP)<0.05)
out.ids2 = which((1-adjP)<0.01)

slide@region.names[out.ids]

# get genomic positions
genome.pos = sapply(slide@region.names,function(x){
  split = strsplit(x," ")[[1]][c(1,3)]
  val = mean(as.numeric(split))
```

```
  return(val)
})
#save the results
outRight  = out.ids
outRight2 = out.ids2
slideOUTright = slide
genome.pos.Right = genome.pos
outRight_names  = slide@region.names[out.ids]
outRight2_names = slide@region.names[out.ids2]
Z_Right = Z

# concatenate results
genome.posCHR3L <- c(genome.pos.Left,genome.pos.INV,genome.pos.Right)
df_CHR3L       <- c(slideOUTleft@df,slideINV@df,slideOUTright@df)

# plot the overall d-fraction
plot(genome.posCHR3L,as.numeric(df_CHR3L),type="l",
ylim=c(-1,1),xlab=c("genome position"),ylab=c("d
fraction"),col="blue",lwd=2)
title(c("Introgression along chromosome 3La"))
abline(v=inversionStart, lty=2)
abline(v=inversionEnd, lty=2)
abline(h=0, lty=2)

#plot the outliers as red points
points(genome.pos.Left[outLeft],as.numeric(slideOUTleft@df[outLeft]),col=
"ORANGE",pch=".",cex=9.0)
points(genome.pos.INV[outINV],as.numeric(slideINV@df[outINV]),col="ORANGE
",pch=".",cex=9.0)
points(genome.pos.Right[outRight],as.numeric(slideOUTright@df[outRight]),
col="ORANGE",pch=".",cex=9.0)
```

```
points(genome.pos.Left[outLeft2],as.numeric(slideOUTleft@df[outLeft2]),col="RED",pch=".",cex=9.0)
points(genome.pos.INV[outINV2],as.numeric(slideINV@df[outINV2]),col="RED",pch=".",cex=9.0)
points(genome.pos.Right[outRight2],as.numeric(slideOUTright@df[outRight2]),col="RED",pch=".",cex=9.0)
```
